# Problems, complications, and reinterventions in 4893 onlay humeral lateralized reverse shoulder arthroplasties: a systematic review (part I—complications)

**DOI:** 10.1186/s10195-021-00592-w

**Published:** 2021-07-08

**Authors:** Francesco Ascione, Alfredo Schiavone Panni, Adriano Braile, Katia Corona, Giuseppe Toro, Nicola Capuano, Alfonso M. Romano

**Affiliations:** 1 Department of Orthopaedic and Trauma Surgery, Ospedale Buon Consiglio Fatebenefratelli, Via A. Manzoni 220, 80123 Napoli, NA Italy; 2 Orthopedics and Sport Medicine Unit, Campolongo Hospital, Salerno, Italy; 3 Dipartimento Multidisciplinare Di Specialità Medico-Chirurgiche Ed Odontoiatriche, Università Degli Studi Della Campania “Luigi Vanvitelli”, Napoli, Italy; 4 Department of Medicine and Health Sciences, Università del Molise, Campobasso, Italy

**Keywords:** Grammont, Results, Revision, Humeral offset, Dislocation, Infection, Radiographic findings

## Abstract

**Background:**

Several modifications to the original Grammont reverse shoulder arthroplasty (RSA) design have been proposed to prevent distinctive issues, such as both glenoid and humeral lateralization. The aim of this systematic review was to determine rates of problems, complications, reoperations, and revisions after onlay lateralized humeral stem RSA, hypothesizing that these are design related.

**Methods:**

This systematic review was performed in accordance with the PRISMA statement guidelines. A literature search was conducted (01.01.2000–14.04.2020) using PubMed, Cochrane Reviews, Scopus, and Google Scholar employing several combinations of keywords: “reverse shoulder arthroplasty,” “reverse shoulder prosthesis,” “inverse shoulder arthroplasty,” “inverse shoulder prosthesis,” “problems,” “complications,” “results,” “outcomes,” “reoperation,” “revision.”

**Results:**

Thirty-one studies with 4893 RSA met inclusion criteria. The 892 postoperative problems and 296 postoperative complications represented overall problem and complication rates of 22.7% and 7.5%, respectively. Forty-one reoperations and 63 revisions resulted, with an overall reoperation rate of 1.7% and overall revision rate of 2.6%.

**Conclusions:**

Problem, complication, and reintervention rates proved acceptable when implanting a high humeral lateralization stem in RSA. The most frequent problem was scapular notching (12.6%), and the most common postoperative complication was scapular stress fracture (1.8%). An overall humeral complication rate of 1.9% was identified, whereas short stems reported no humeral fractures or stem loosening. Infections (1.3%) proved to be the most common reason for component revision, and instability had a complication rate of 0.8%.

**Level of evidence:**

Systematic review IV

## Introduction

Grammont-style reverse shoulder arthroplasty (RSA) has been reported to provide satisfactory clinical results for several shoulder pathologies [1–4], but this design has been found to have several drawbacks inherent to altered joint biomechanics. Firstly, excessive medialization may lead to a slackening of any intact cuff, which could contribute to undesirable instability, poor restoration, and weakness of internal and external rotation[1, 5]. Secondly, the contour of the shoulder is somewhat altered, and the physiological wrapping angle of the deltoid decreases from 48° to 8°, contributing to instability in association with an insufficient cuff [6–8]. Finally, the 155° neck-shaft angle (NSA) and glenoid medialization led to peripheral impingement and high rates of scapular notching [1, 4] with the potential for polyethylene wear, glenoid loosening, osteolysis, and tuberosity resorption [8, 9].

Studies have shown that bone lysis, component loosening, and overall complications frequently regard the humeral side (1.5–10%) [1, 10–13].

Subsequent RSA designs have attempted to address some of these issues by providing more lateralized reconstruction. Modifications of the stem design have been proposed: (I) a change in the NSA to 145° or 135° to decrease scapular notching, (II) curved and short stems to preserve bone stock and tuberosities, and (III) onlay systems to facilitate conversion from an anatomic arthroplasty. These changes translate into humeral lateralization, which presents several advantages. It restores a more natural anatomical position of the humerus and therefore of the lesser and the greater tuberosities, which improves the length/tension of the remaining cuff [1, 14, 15], thus increasing compressive forces on the joint and improving stability [15]. A more lateral position of the greater tuberosity increases the abductor lever arm and the wrapping angle of the deltoid [9], which enhances compressive forces [6, 16, 17].

Medialized implants are now a minority, but the ideal amount of global lateralization and the ideal contribution from the glenoid or the humerus remain unknown. Werthel et al. [18] provided a clear definition of humeral lateralization and values of lateralization in the most commonly used, currently available RSA implants. They concluded that restoring anatomical insertion of remaining cuff, deltoid wrapping angle, and greater tuberosity lateralization corresponds to high humeral offset implants. Lateralization in both the humerus and the glenoid combines the beneficial effects of each lateralization, but the risk is that excessive lateralization may be problematic in smaller patients or in the presence of soft-tissue contractures; resultant joint overstuffing may lead to poor motion, polyethylene wear [15, 16], difficulty in joint reduction, nerve stretching, difficulty with the repair of the subscapularis [14, 19], acromial impingement, and/or fractures [20].

The purpose of the present study, therefore, was to perform a systematic review of the published literature to determine the overall rates of problems, complications, reoperations, and revisions after onlay lateralized humeral stem RSA. It was hypothesized that emerging reinterventions, problems, and complications are peculiar to new design prostheses and that their significance differs from that of Grammont-style RSA. In this part (part I), a systematic review about complications was conducted.

## Materials and methods

This systematic review was conducted according to the guidelines of the preferred reporting items for systematic review and meta-analysis (PRISMA) statement (http://prisma-statement.org).

### Search strategy

A systematic review of the available literature was conducted using synonymous or related expressions for the terms “reverse shoulder arthroplasty,” “reverse shoulder prosthesis,” “inverse shoulder arthroplasty,” “inverse shoulder prosthesis,” “problems,” “complications,” “results,” and “outcomes” in several combinations. The following databases were assessed: PubMed, Cochrane Reviews, Scopus, and Google Scholar. The search was performed from 1 January 2000 to 14 April 2020. All peer-reviewed journals were considered; randomized controlled trials (RCTs), prospective trials (PRO), and retrospective studies (RE) were included. The search was limited to papers in the English language. Two authors (A.B. and G.T.) independently screened the titles and abstracts, and subsequently performed a full-text selection of the articles resulting from the search. All references of the included studies were subsequently searched manually to identify any additional articles that may not have been captured in the initial search. In the event of disagreement, a consensus was reached by discussion, with the intervention of the senior author when necessary (F.A.).

### Study selection

For the aforementioned aim, the implants included derived from the study of Werthel et al. [18], with prostheses of minimum 10 mm humeral lateralization compared with Grammont-style RSA, resulting in a 10–14.7 mm lateral offset range, 135–145° NSA and all onlay designs.

To be considered eligible for inclusion, studies needed to (1) include patients who had undergone an onlay humeral lateralized RSA; (2) report data on problems, complications with declared implants; (3) be a published RCT, RE study or PRO trial.

Studies were excluded if (1) the articles were not in English; (2) it was impossible to extrapolate or calculate the necessary data from the published results; (3) they were a review article or technical note; (4) they involved animal experiments or in vitro trials; (5) they focused exclusively on acute fractures, revisions, or tumor surgery series; (6) there was heterogeneous use of Grammont and humeral lateralized arthroplasties in a single cohort.

### Level of evidence

The Oxford Levels of Evidence as produced by the Oxford Centre for Evidence-Based Medicine were used to categorize methodological quality (http://www.cebm.net/ocebm-level-of-evidence/). This tool classifies systematic randomized clinical trials and inception cohort studies as Level II evidence, cohort studies or control arm of randomized trials as Level III evidence, and case series or case–control studies or poor-quality prognostic cohort studies as Level IV evidence.

### Methodological quality assessment

Methodological evaluation was performed according to the MINORS evaluation [21], which was specifically created to evaluate the quality of nonrandomized surgical studies. The checklist includes 12 items, with the last four specific to comparative studies. Scoring was as follows: 0, not reported; 1, reported but poorly done and/or inadequate; and 2, reported, well done, and adequate. The highest overall score was 16 for noncomparative studies and 24 for comparative studies.

### Data extraction

Two authors (A.B. and G.T.) extracted data from all selected original articles; this procedure was repeated by another author (K.C.). If no agreement could be reached, the senior author was consulted (F.A.). Data were extracted from each article included and entered into a spreadsheet for analysis. Pertinent extracted information included the following: author, date and journal of publication, study design and level of evidence, patient demographics (number of shoulders enrolled, gender, age, and follow-up), the prosthetic implant used, the surgical approach, the diagnosis leading to RSA, intraoperative complications, and postoperative problems/complications, from all studies systematically using a table template. Definition of complication was based on a previously published review [22], with certain modifications (Table 1). A 0% rate of complication was reported whenever the authors stated that none of their patients had that problem or complication, whereas the value was left as unreported when authors did not mention the problem or complication.

**Table 1:** Definitions of problems, complications, reoperations, revisions

	Definition	Examples
Problems	Intraoperative or postoperative event that was not likely to affect the patient’s final outcome	Radiographic scapular notching, hematomas, glenoid or humeral nonprogressive radiolucent lines, heterotopic ossification, scapular spurs, chronic pain and/or stiffness, intraoperative dislocations, intraoperative cement extravasation, or other radiographic findings of the humerus or the eventual glenoid graft
Complications	Any intraoperative or postoperative event that was likely to have a negative influence on the patient’s final outcome	Fractures, infections, dislocations, nerve palsies, aseptic loosening of humeral or glenoid components, prosthetic component disassociations, or glenoid graft failures
Reinterventions		
Reoperations	Intervention requiring any return to the operating room for any reason relating to the shoulder, without replacing humeral/glenoid components	PE insert exchanges, ORIF, debridement, arthroscopy, tendon transfers
Revisions	Surgeries with total or partial exchange or removal of the components	Stem exchanges, glenoid baseplate/glenosphere exchanges, humeral spacers

*PE* polyethylene insert; *ORIF* open reduction internal fixation

## Results

### Literature search

**Fig. 1:**
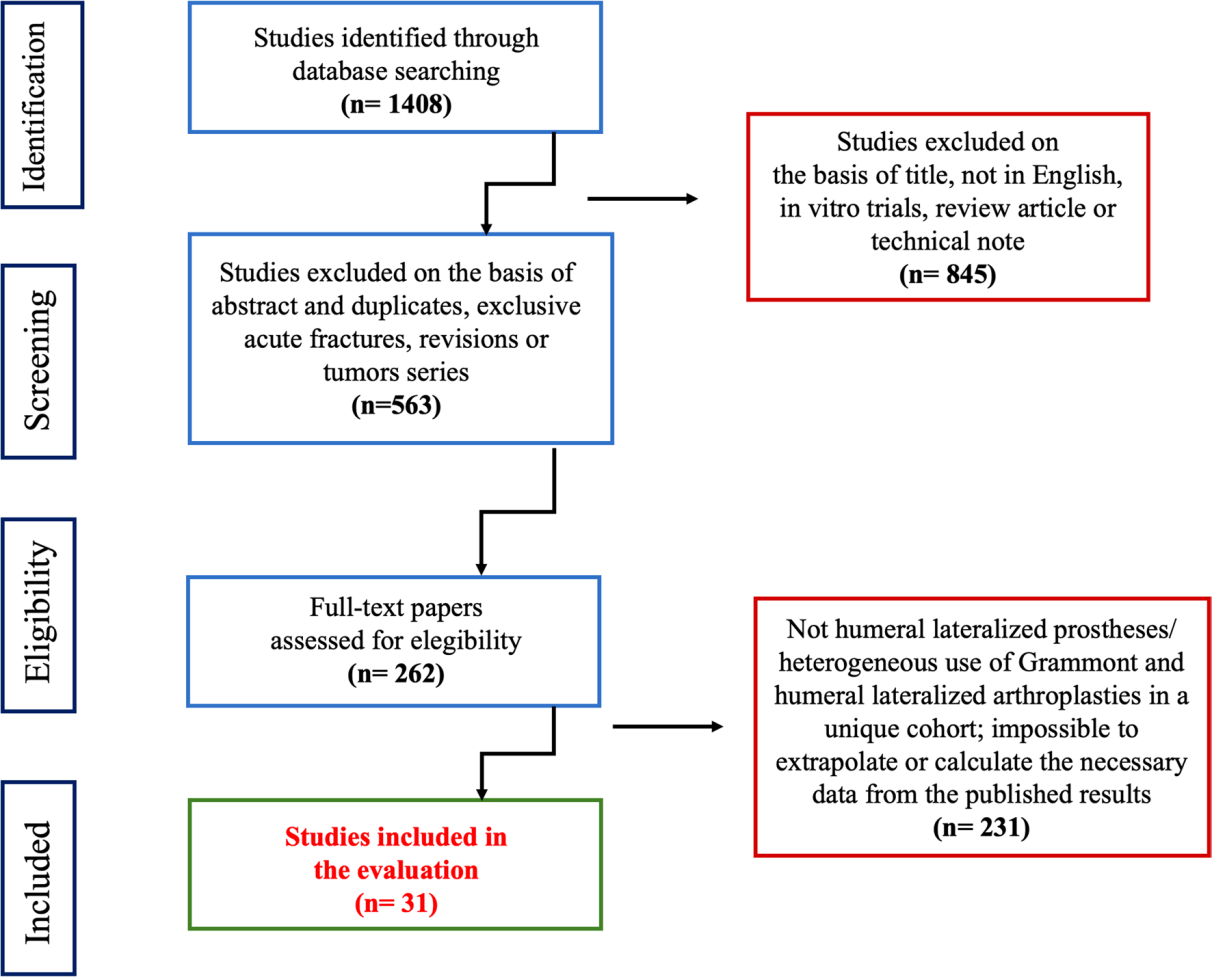
Studies collection and inclusion process

The initial search resulted in 1408 articles. The abstracts of these studies were reviewed to determine the applicability to the present study as determined by the inclusion and exclusion criteria, including a worksheet adapted from evidence-based guides (Fig. 1).

A total of 4893 RSA were included from 31 studies [14, 19, 20, 23–50].

Average MINORS scores were 13.7/16 for noncomparative studies and 19.4/24 for comparative studies, thus determining acceptable study quality level.

### Demographic data, surgical technique, and etiology

Demographics of the reviewed cohort, and follow-up, including study design, level of evidence, surgical information, and etiology, are summarized in Table 2. Twenty-five studies declared the gender of patients, and mean postoperative follow-up was 38.6 months.

**Table 2:** Demographic data and study characteristics

First author	Year	Level of evidence	RSA cases	Subgroups	Implanted RSA	Approach	Cemented	Age (years)	Range (± SD)	Follow-up (months)	Range (± SD)	Males	Females
Franceschetti	2020	III	59	Not BIO 29; BIO 30	Aequalis Ascend Flex (Tornier–Wright)	DP	N/S	69.7; 70	N/S (± 9.9); N/S (± 7.8)	24.6; 25.2	N/S (± 1.1); N/S (± 1.6)	10; 13	20; 16
Simovitch	2019	III	324	Notch 47; not Notch 277	Equinoxe (Exactech)	DP	50	72.0	38–89 (± 7)	75.1	60–132 (± 16.9)	N/S	N/S
Franceschetti	2019	III	84	SSR 44; not SSR 40	Aequalis Ascend Flex (Tornier–Wright)	DP	N/S	70.18; 69.71	N/S (± 10.63); N/S (± 6.14)	15.9; 16.92	N/S (± 1.29); N/S (± 1.92)	10; 11	34; 29
Choi	2019	IV	38		Comprehensive reverse (Biomet)	DP	0	73	63–83	24	12–53	6	32
Aibinder	2019	IV	65		Comprehensive micro stem (Zimmer Biomet)	DP	0	N/S	N/S	45.6	N/S	N/S	N/S
Raiss	2019	IV	77		Aequalis Ascend Flex (Tornier–Wright)	DP	N/S	72	50–91	28	24–48	N/S	N/S
Matsuki	2018	III	552	Small 130; average 384; tall 38	Equinoxe (Exactech)	N/S	N/S	74; 72; 72	50–85; 52–93; 59–84	41; 37; 37	24–94; 24–97; 24–98	4; 178; 37	126; 206; 1
Merolla	2018	III	38		Aequalis Ascend Flex (Tornier–Wright)	DP	3	74.7	55–91	29.1	24–31	13	25
Ascione	2018	IV	485	Sc Fr 21; not Sc Fr 84	Aequalis Ascend Flex (Tornier–Wright)	DP	N/S	72.6; 72.3	N/S (± 7.1); N/S (± 7.6)	16.3; 16.5	6.5; 9.1	6; 24	15; 60
Alentorn-Geli	2018	III	16	All B2 glenoids	Comprehensive reverse (Zimmer Biomet)	DP	N/S	72.5	N/S (± 5.4)	35.1	N/S (± 14.2)	N/S	N/S
Werner BC	2018	III	109	SSR 71; not SSR 38	Aequalis Ascend Flex (Tornier–Wright)	N/S	N/S	71.1; 70.7	N/S (± 10.7); N/S (± 8.6)	24; 25.2	N/S (± 18); N/S (± 18)	28; 15	43; 23
Zilber	2018	IV	35		Aramis (3S ortho)	DP	0	73	45–86	24	N/S	7	28
Werner BS	2017	IV	56		Aequalis Ascend Flex (Tornier–Wright)	DP	9	74.6	56–91	30.1	24–44	15	41
Mollon	2017	III	476		Equinoxe (Exactech)	N/S	N/S	72.5	53–90	38	22–93	164	312
Romano	2017	IV	112		Equinoxe (Exactech)	DP	0	72.2	60–87	29.2	12–36	29	83
Kennon	2017	III	318	Bp sup screw 206; Bp inf screw 112	Equinoxe (Exactech)	DP	N/S	65.3; 65	N/S (± 11); N/S (± 10.8)	N/S	N/S	85; 44	121; 68
Ascione	2017	IV	100		Aequalis Ascend Flex (Tornier–Wright)	DP	20	73.4	55–91	32.6	24–44	28	72
Lädermann	2017	II	35		Aequalis Ascend Flex (Tornier–Wright)	DP 18, SSCS 17	N/S	78	N/S (± 7)	18	12–46 (± 11)	8	27
Schnetzke	2017	IV	25		Aequalis Ascend Flex (Tornier–Wright)	DP	0	N/S	N/S	25	20–35	5	20
Vourazeris	2017	III	202	SSR 86; not SSR 116	Equinoxe (Exactech)	DP	N/S	71.6; 71.1	N/S	39.6; 37.2	N/S	N/S	N/S
Grubhofer	2017	IV	44		Reverse anatomical shoulder system (Zimmer)	N/S	31	68	30–86	46	24–108	32	12
Hurwit	2017	III	40		Comprehensive reverse (Biomet)	N/S	N/S	68.6	N/S (± 7.6)	32.2	N/S (± 14.9)	16	24
Friedman	2017	III	591	SSR 340; not SSR 251	Equinoxe (Exactech)	N/S	N/S	72.2; 72.9	N/S	37.3; 35.7	N/S	119; 105	221; 146
Mollon	2016	III	297		Equinoxe (Exactech)	N/S	N/S	72	50–88	39	24–91	99	191
Jones	2016	IV	44	All glenoid autograft; allograft	Equinoxe (Exactech)	DP	N/S	69.1	N/S (± 7.4)	40.6	16	20	24
Dezfuli	2016	III	36	FrS 24; HA Rev 12	Equinoxe (Exactech)	DP	36	73; 66; 66	N/S	36; 34; 24	N/S	4; 2; 2	9; 10; 9
Katz	2016	IV	140		Arrow (FH Orthopedics)	25 DP, 115 SL	34	72	52–90 (± 6.91)	45	24–120	34	100
King	2015	III	83		Equinoxe (Exactech)	DP	32	72	55–93	42	N/S	32	51
Gilot	2015	III	292	Cem 177; 115 UnC	Equinoxe (Exactech)	DP	177	N/S	N/S	39.76	N/S	N/S	N/S
Giuseffi	2014	IV	44		Comprehensive reverse (Biomet)	DP	0	76	59–92	27	24–40	15	29
Valenti	2011	IV	76		Arrow (FH Orthopedics)	SL	6	73	52–90	44	24–60	18	58
Total	…	…	4893	SSR 541/986	Equ. 68%, AFl 19.6%, Com 6.4%, Arr 4.4%, Inv 0.9%, Ara 0.7%	2593 DP + 191 SL/2784	398/1508	4511	…	4195	…	1238	2286
Mean/%				SSR 54.9%		DP: 93.1%; SL: 6.9%	26.4%	71.5	…	38.6	…	35.1%	64.9%

*RSA* reverse shoulder arthroplasty; *SD* standard deviation; *Notch* scapular notching; *BIO* bony increased-offset reverse shoulder arthroplasty; *Notch* notching; *SSR* subscapularis tendon repaired; *Sc Fr* scapular fractures; *Bp sup screw* metaglene baseplate superior screw used; *Bp inf screw* metaglene baseplate inferior screw used; *FrS* fracture sequelae; *HA Rev* hemiarthroplasty revisions; *Cem* cemented stem; *UnC* uncemented stem; *DP* deltopectoral; *SL* superolateral; *SSCS* subscapularis and deltoid sparing; *N/S* not stated; *Equ* Equinoxe (Exactech); *AFl*, Aequalis Ascend Flex (Tornier–Wright); *Com* comprehensive reverse (Biomet); *Arr* Arrow (FH Orthopedics); *Inv* reverse anatomical shoulder system (Zimmer); *Ara* Aramis (3S ortho)

Subscapularis repair was studied in four articles [14, 19, 29, 48]. Sixteen studies included intraoperative stem cementation data; of 1508 cases, 398 stems were cemented (26.4%), and six studies reported press-fit stems [23, 26, 31, 44, 45, 50] only.

Four papers reported clear information regarding glenoid grafts: Franceschetti et al. [28] compared 29 standard glenoid RSA with 30 BIO-RSA [51], with glenoid lateralization as the single aim; Merolla et al. [40] and Ascione et al. [25] presented heterogeneous groups of shoulders, with both BIO-RSA and angled BIO-RSA [52] in the correction of severe glenoid defects and retroversion (20 and 53 glenoid grafts, respectively); Jones et al. [34] reported 44 shoulders with severe glenoid bone loss, both in primary implants and in revision surgeries, treated with RSA and glenoid autografts or allografts.

The indication for implanting an RSA was stated in 28 studies (4100 cases), but 12 studies did not state the number of cases for each etiology, and in one, some cases were unstated [47], leaving a total of 1170 arthroplasties with definite pathologies. The most frequent surgical indications were cuff tear arthropathy (CTA) in 587 shoulders (50.2%). Details of the analyzed study etiologies are reported in Table 3.

**Table 3:** Etiologies

First author	Year	CTA	OA	FrS	MRCT	Rev	IA	AvN	AcF	InstA
Franceschetti	2020	38								
Simovitch	2019									
Franceschetti	2019									
Choi	2019	30	3		5					
Aibinder	2019	33	25	1			4	2		
Raiss	2019									
Matsuki	2018	258	207		61		11	9		
Merolla	2018	59								
Werner BC	2018									
Zilber	2018	19	10	3				1	2	
Werner BS	2017	44		5	5		2			
Mollon	2017									
Romano	2017									
Kennon	2017									
Ascione	2017	46	28	8	14		2			2
Lädermann	2017	10		8	17					
Schnetzke	2017	17	2	5						
Vourazeris	2017									
Grubhofer	2017			44						
Hurwit	2017									
Friedman	2017									
Mollon	2016									
Jones	2016									
Dezfuli	2016			24		12				
Katz	2016									
King	2015									
Giuseffi	2014	33		2			3	6		
Valenti	2011			15		25	2		6	2
Total		587	275	115	102	37	24	18	8	4
%		50.2	23.5	9.8	8.7	3.2	2.1	1.5	0.7	0.3

Gray cells: studies that did not declare number of cases for each etiology

Alentorn-Geli [2], Ascione [6], and Gilot [22] did not declare etiologies

*CTA* cuff tear arthropathy; *OA* glenohumeral osteoarthritis; *FrS* fractures sequelae; *MRCT* massive rotator irreparable cuff tears; *Rev* revisions; *IA* inflammatory arthritis; *AvN* avascular necrosis of the humeral head; *AcF* acute fractures; *InstA*, instability arthropathy

## Results

Of the 3926 cases, the 296 postoperative complications represented an overall rate of 7.5%. Ten studies reported intraoperative complications of 665 arthroplasties: three humeral fractures, three glenoid fractures, one axillary artery injury, making a total of seven cases (1.1%).

Table 4 illustrates the postoperative complications of the present review. Acromial and scapular spine fractures were the most common postoperative complication, with a mean incidence of 1.8% (77 fractures of 4393 cases). With classification according to Levy [53] (2657 RSA), a total of 14 acromion fractures (type I) and 41 spine fractures (type II–III) were reported (74.5%).

**Table 4:** Postoperative complications

Postoperative complications	Cases (no.)	%
Instability	29	0.8
Acromion and scapular spine fractures	77	1.8
Type I	− 14	
Type II–III	− 41	
Humeral fractures	50	1.4
Infections	47	1.3
Aseptic glenoid loosening	39	1.1
Humeral loosening	19	0.5
Glenoid/humeral disassembly	16	
Neurologic complications	12	0.4
PE wear	2	
Unspecified implant failures	2	
Pulmonary embolism	2	0.05
Glenoid graft failure	2	
Hematoma	1	
Draining axilla folliculitis	1	
Shoulder pseudoparalysis	1	
Total	296	7.5

*PE* polyethylene insert

Fractures of the scapular spine, however, compromised the final outcome in analyzed shoulders [20, 36]. This complication was more frequent in certain studies with implanted Equinoxe Reverse and Ascend Flex Reverse prostheses (3–5%) [20, 25, 36, 40, 41, 46], but the focus on scapular fractures in some studies and in others, both arthroplasties with lower rates, led to confusing conclusions.

All postoperative humeral fractures (50 cases of 3590) were associated with a traumatic event in 25 studies, resulting in 1.4% incidence. Twenty-nine cases (58%) were reported in press-fit stems, and one case of fracture of the greater tuberosity was included [35].

No cases were reported in short-stem studies (Ascend Flex and Comprehensive Micro Stem) [14, 20, 23, 25, 28, 38, 40, 43, 45, 49], whereas the Equinoxe RSA was implanted in the majority of cases (48 fractures).

Humeral loosening (19 cases of 3882) was reported in 26 different studies, with a mean incidence of 0.5%. These were only revised in cases of clinical impact (six cases). Similar to humeral fractures, 16 studies including 1142 arthroplasties, all short stems, did not report any loose humeral component.

Aseptic glenoid loosening (39 cases of 3448) was not reported as being related to a progression of inferior scapular notching, a rate of 1.1% (23 studies). One case of glenoid loosening was a consequence of traumatic glenoid fracture [26] and two subsequent to graft failure [34].

Instability (29 dislocations out of 3634 cases) had a mean incidence of 0.8% in 25 studies concerning complications. A total of seven studies including 631 RSA did not record any dislocation [24, 26, 27, 32, 41, 45, 50]. The role of subscapularis repair in instability was investigated in four studies[14, 19, 29, 48], with a total of 986 arthroplasties: seven dislocations occurred in 445 nonrepaired subscapularis (1.6% rate) and two dislocations in 541 repaired tendons RSA (0.4% rate).

Neurologic complications were mentioned in 12 shoulders of the 3114 examined, with an average incidence of 0.4%. They included four brachial plexus, three axillary, four unspecified brachial plexus/axillary nerve palsies, and one case of persistent limb numbness.

On further investigation, it was found that two cases of brachial plexus palsy had required prosthesis components revision, and one axillary palsy had recovered conservatively in 15 months.

Of the 3525 RSA included, 47 cases of infection (1.3%) were reported in 24 studies.

Rare complications included ten cases of unspecified glenoid/humeral disassembly; six humeral disassembly; two PE wear, both in two studies that considered two different generations of the Arrow Reverse [35, 47], which were attributable to mechanical failure in the first-generation prostheses, successfully replaced in 2005; two cases of unspecified implant failure [39]; and two cases of pulmonary embolism (0.05%) [29, 39].

## Discussion

The present review demonstrated acceptable complications rates, when implanting a high humeral lateral offset (range 10–14.7 mm), 135–145° NSA and onlay system RSA, compared with Grammont-style designs [18]. The global rate for complications after onlay humeral lateralized RSA was 7.5%, at 3 years mean follow-up. To our knowledge, no studies in literature have thoroughly investigated the topic of this systematic review.

The principal finding was that prostheses with a lateralized humeral stem resulted in lower rates in the distinctive complications of Grammont-style RSA, such as instability (0.8%), which was reported as much higher in previous literature[1–4, 10–13, 22, 54–57].

In the past, the safest methods of preventing problems/complications after RSA, such as scapular notching and dislocations, were considered to be inferior positioning/tilting of the glenoid baseplate and larger-size glenospheres, and the use of bone or metal increased offset glenoids [51, 56, 57]. Analogous to Grammont RSA, the rate of aseptic glenoid loosening is not reported to be a major problem in the present review, with a prevalence of 1.1%. Preoperative assessment of glenoid bone stock and careful planning for optimal positioning of the metaglenoid remain important in preventing loosening. Previously, glenoid loosening had been reported at a mean 2.5% prevalence [11, 13, 22, 56], and even up to 12% in certain glenoid lateralized prostheses [2, 55–57].

More recently, the attention has switched to lateralizing the humeral side, achieved by various means with several aforementioned advantages. Firstly, the stem may be modified from straight to curved [25]. Secondly, the humeral bearing may rest on the humeral osteotomy, the onlay system, lateralizing the humerus by displacing the stem away from the glenosphere, and all implants evaluated in this study had an onlay design, thus preserving metaphyseal bone, ensuring ease of conversion and providing additional modularity of the insert for NSA [45]. Modification of the NSA from 155° to 145° or 135° has been described as a cause and/or a means of humeral lateralization, with no increase in instability [58].

In contrast to manifestations of humeral stress shielding, stem loosening as well as migration (0.5%) not only contributes to shoulder dysfunction but may also be a contributing factor in the failure of the prosthesis. Although loose stems were not stated as cemented or not, 68% of cases were pinpointed in studies with a consistent presence of cemented stems, whereas six studies of press-fit RSA reported no cases of humeral loosening, suggesting a higher risk of failure in cemented stems, as previously reported [59]. Sixteen studies including 1142 arthroplasties, all implanted short stems, reported no loose humeral component, confirming that proximal humeral bone preservation is crucial in preventing humeral loosening.

Humeral subsidence has been proven to achieve 2–4% occurrence in Grammont RSA implants since, in protecting the glenoid from stress, constraint and torsional forces frequently lead to changes on the humeral side [10, 12, 22, 24, 45, 53, 54, 59].

Conservative treatment is proposed for shoulders that have not manifested a significant worsening of functional scores, severe radiographic loosening and/or migration, or proximal bone loss; in the present study, only 30% of loose stems underwent revision, a viable option to achieve enhanced implant stability. However, the second cause of RSA revision proved to be humeral or glenoid component loosening.

An overall humeral complication rate of 1.9% was identified in the present review, varying only slightly from previously published reports (1.5–10%) [11, 12, 22, 24, 59], but these also included short-stem studies (Ascend Flex and Comprehensive Micro Stem), which demonstrated a significant decrease in the risk of humeral fractures and stem loosening, reporting no cases of shoulders affected by these complications.

All postoperative humeral fractures (1.4%) were traumatic, 58% occurring in press-fit stems and 96% in Equinoxe Reverse, suggesting an association of weaker osteoporotic bone and surgical technique factors. Campbell et al. [60] suggested that over-reaming of the endosteal diaphysis for cement implantation can lead to periprosthetic fractures through increased hoop stresses. Other reported risk factors for fractures are related to operative technique (oversized implants, poor surgical exposure, over-rotated arm during stem insertion). Fractures were generally treated conservatively in 74% of cases, and in transverse or spiral fractures with minimal displacement, splint immobilization can ensure consolidation in 3–6 months.

The most common postoperative complication was scapular fracture (1.8%), the majority of which were spine type II–III fractures (75%). Where the cause of fracture was stated, all cases were described as atraumatic stress fractures: lateralization in both the humerus and the glenoid combines the beneficial effects of both glenoid and humeral lateralization, but the risk is the production of excessive stress on elderly patients’ osteoporotic bone [61], especially in significant humerus lengthening [49].

Acromion/spine fractures were particularly prevalent in Equinoxe and Ascend Flex prostheses (from 3% to 5%) [20, 25, 36, 40, 41, 46], a fact which was noted in detail in two studies that thoroughly investigated fractures.

However, this did not appear to be related to a particular prosthetic design but, rather, was equally distributed among studies that pointed out scapular fractures in onlay lateralized stems. Other studies with both arthroplasties, however, reported lower rates.

As pinpointed by Haidamous et al. [62], the risk might be related to an association of lateralization and distalization due to onlay designs. The authors concluded that increased postoperative distalization is associated with an increased risk of scapular spine fracture following RSA. An onlay humeral stem design resulted in a 10 mm increase in distalization compared with an inlay humeral stem, and a 2.5 times increased risk of fracture. On the other hand, lateralization did not appear to increase the risk.

What is more, incidence of spine fractures may be underreported because it is difficult to ensure that the included studies have not missed any fractures (they are relatively rare by most estimates, are challenging to diagnose with plain radiographs, occasionally are minimally symptomatic, and may be a cause of unidentified pain) [63]. Grammont-design implants reported inhomogeneous prevalence rates from 1% to 10% because of confusing factors such as small sample size, use of different prostheses in the same series, progressive modifications of RSA design, use of different classification, or inclusion of preoperative acromial insufficiencies (1–10%) [11, 22, 53, 63], although it appears that a certain rate of acromion fracture was reported in Grammont series and higher rates of spine fractures in lateralized design RSA [20, 36]. Scapular spine fractures lead to inferior functional and active mobility outcomes, regardless of the treatment modality.

Infection (1.3%) was reported to be less frequent in RSA than in knee or hip arthroplasty[64], with a trend toward lower infection than previously reported rates (3–6%) [11, 13, 22, 65]. *Cutibacterium acnes* and *Staphylococcus aureus* were the most frequently involved pathogens: these bacteria are typically responsible for late, chronic, relatively low-grade infections, colonizing loose prostheses and, exceptionally, acute postoperative infection and are likely to be present in more revision cases than generally suspected [11, 66]. Treatment of acute infection using antibiotics and debridement with retention, irrigation, and suction, complemented by intravenous antibiotics is a choice for infections with symptoms at less than 3 weeks in a stable prosthesis and no growth on preoperative cultures [65]. This regimen is ineffective in chronic or late infections requiring revision, with no clear tendency to use a one-stage or two-stage procedure. However, infections remained the most common reason for component revision of the studies included, and the incidence of infection may increase at longer follow-up periods.

Postoperative dislocations subsequent to RSA achieved a prevalence of 0.8%, suggesting that one of the main purposes of humeral lateralized design, the increase of prosthetic construction stability, had been obtained. Grammont-style RSA large series and reviews showed higher instability incidence, ranging from 3% to 14% [2–4, 11, 13, 22, 55, 67], although studies included only glenoid lateralized designs [57].

Biomechanically, lack of compressive forces between the glenosphere and humeral socket are the main parameters associated with instability, consequent to a loss of tension of the deltoid and the remaining cuff associated with proximal bone resorption in late dislocations. Small glenoid size, the deltopectoral approach, poor subscapularis muscle condition, revision surgery, younger male patients, association with scapular notching, resorbed tuberosities/proximal bone loss, and inadequate humeral length or version were all reported to be factors relating to higher rates of instability [11, 22, 65, 67, 68].

Adequate subscapularis repair has been recognized as one of the principal means of preventing instability in Grammont RSA through the deltopectoral approach [1, 47, 48]. Despite this novel design, the role of subscapularis continues to play a role in RSA stability: four dedicated studies [14, 19, 29, 48], with a total of 986 lateralized prostheses, showed a significant increase in dislocations in nonrepaired subscapularis RSA (1.6%) compared with repaired tendon groups (0.4%).

Although the treatment of prosthetic instability can be conservative, revision surgery may be required in recurrent dislocations and in those occurring in the first few months [67].

Fortunately, neurologic injuries were very rare (0.4%) and had an effect only in cases of incomplete recovery: only one case of the 3114 RSA included led to humeral component revision to resolve a brachial plexus palsy. Other brachial plexus/axillary nerve palsies recovered spontaneously, and no reports of radial or ulnar nerve problems were found.

Postoperative glenoid or humeral disassembly and polyethylene disassociations were infrequent and only mentioned as a problem related to the design of the Arrow prosthesis used before 2005, which was resolved after a new implant design [35, 47].

### Limitations and strengths

Our investigation is an up-to-date systematic review of the literature, which considers implants categorized as humeral onlay lateralized design as compared with original Grammont RSA [18]. To date, no studies have thoroughly investigated this particular design in a systematic review.

However, we have identified some study limitations. Firstly, given that almost all the studies included were therapeutic case series, this study corresponds to an indirect level III–IV of evidence, and further comparative studies are clearly needed to investigate at level I–II of evidence.

Second, the definition of a complication differs significantly among the studies. This may decrease the accuracy of the comparison between the results of this study and those in existing literature. This issue does not affect the accuracy of the analysis in this study, as special attention was paid when collecting data from all the studies included to adequately classify complications according to the aforementioned definitions and provide adequate homogenization.

Thirdly, we intentionally excluded studies regarding revision cases only or only proximal humerus acute fractures; this may result in underestimated rates of complications, but this decision [3, 11, 13, 22] was taken on the basis of the purpose of analyzing this particular design in the most common indications for RSA, and high rates of complications are mainly related to revision/fracture surgery, and not the RSA itself.

Finally, there are a huge number of factors that can influence the rates considered, and these are not well controlled in the existing evidence: length of follow-up, surgeon’s experience, different rehabilitation protocols, type of glenosphere (eccentric or concentric, medialized or lateralized), humeral version, degree of bone stock and glenoid erosion, use of cement, or previous surgeries.

## Conclusion

Complication rates may be regarded as acceptable, 7.5%, when implanting a humeral lateralized stem, 135–145° NSA and onlay RSA. Low overall rates of instability (0.8%) were reported; the most common postoperative complication proved to be scapular stress fracture (1.8%), suggesting an increase in the force acting through the deltoid.

A total rate of 1.9% humeral complications was identified, whereas short stems demonstrated no humeral fractures or stem loosening.

## Data Availability

Not applicable.
